# The GABA_A_ receptor α2 subunit gene (GABRA2) is associated with alcohol-related behavior

**DOI:** 10.1186/2050-6511-13-S1-A8

**Published:** 2012-09-17

**Authors:** Dubravka Švob Štrac, Gordana Nedić, Matea Nikolac, Korona Nenadić Šviglin, Dorotea Mück-Šeler, Nela Pivac

**Affiliations:** 1 Division of Molecular Medicine, Ruđer Bošković Institute, 10000 Zagreb, Croatia; 2 Center for Alcoholism and other Addictions, Psychiatric Hospital Vrapče, 10000 Zagreb, Croatia

## Background

γ-Aminobutyric acid type A (GABA_A_) receptors, the major inhibitory neurotransmitter receptors in the brain, are implicated in the acute and chronic effects of alcohol, including tolerance, dependence and withdrawal. Various polymorphisms in the gene encoding the GABA_A_ receptor α2 subunit (GABRA2) have been associated with alcoholism and with antisocial behavior in different populations of European ancestry. As early onset of alcoholism often reflects greater severity, including a higher risk for recurrence, comorbid antisocial personality disorder and conduct disorder, Cloninger’s classification distinguishes type II alcoholism with an early onset, elevated levels of antisocial behavior and delinquency, from the type I alcoholism with a late onset, neurotic symptoms and minimal criminality.

## Methods

Genotyping of GABRA2 polymorphisms (rs567926, rs279858 and rs9291283) was performed in samples of 355 alcoholic patients of Croatian origin (280 males and 75 females) using TaqMan Real-Time allelic discrimination technique after extraction of DNA from whole blood. The results of allelic and haplotypic analysis were compared between alcohol-dependent subjects with a combination of early onset of alcohol abuse and presence of aggressive behavior corresponding to type II alcoholism subgroup, and individuals with the late onset of alcohol abuse and without aggression corresponding to type I alcoholism subgroup, according to Cloninger.

## Results

Cloninger’s Type I and Type II alcohol-dependent patients did not differ significantly in the frequency of the genotypes and alleles for rs567926, rs279858 or rs9291283. However, the G-T-G haplotype was more often present in the alcohol-dependent subjects with early onset of alcohol abuse and aggressive behavior, corresponding to the Cloninger type II alcoholism subgroup (χ^2^ = 6.102, p = 0.013).

## Conclusions

Our results revealed a haplotypic association between the GABRA2 gene and a more severe form of alcoholism, characterized by the early onset of alcohol abuse and presence of aggressive behavior. These findings support an important role of GABA_A_ receptors in the susceptibility to alcoholism and highlight them as potential targets for novel therapeutics in the treatment of alcohol dependence.

